# Survival of cancer patients treated with mistletoe extract (Iscador): a systematic literature review

**DOI:** 10.1186/1471-2407-9-451

**Published:** 2009-12-18

**Authors:** Thomas Ostermann, Christa Raak, Arndt Büssing

**Affiliations:** 1 Center for Integrative Medicine, Faculty of Medicine, University of Witten/Herdecke, Gerhard-Kienle-Weg 4, 58239 Herdecke, Germany

## Abstract

**Background:**

In Europe, extracts from *Viscum album *(VA-E), the European white-berry mistletoe, are widely used to treat patients with cancer.

**Methods:**

We searched several databases such as Cochrane, EMBASE, NCCAM, NLM, DIMDI, CAMbase, and Medline. Inclusion criteria were controlled clinical studies on parameters associated with survival in cancer patients treated with *Iscador*. Outcome data were extracted as they were given in the publication, and expressed as hazard ratios (HR), their logarithm, and the respective standard errors using standard formulas.

**Results:**

We found 49 publications on the clinical effects of *Iscador *usage on survival of cancer patients which met our criteria. Among them, 41 studies and strata provided enough data to extract hazard ratios (HR) and their standard errors (*Iscador *versus no extra treatment). The majority of studies reported positive effects in favour of the *Iscador *application. Heterogeneity of study results was moderate (I^2 ^= 38.3%, p < 0.0001). The funnel plots were considerably skewed, indicating a publication bias, a notion which is corroborated by statistical means (AC = -1.3, CI: -1.9 to -0.6, p <= 0.0001). A random effect meta-analysis estimated the overall hazard ratio at HR = 0.59 (CI: 0.53 to 0.66, p < 0.0001). Randomized studies showed less effects than non-randomized studies (ratio of HRs: 1.24, CI: 0.79 to 1.92, p = 0.35), and matched-pair studies gave significantly better results than others (ratio of HRs: 0.33; CI: 0.17 to 0.65, p = 0.0012).

**Conclusions:**

Pooled analysis of clinical studies suggests that adjuvant treatment of cancer patients with the mistletoe extract *Iscador *is associated with a better survival. Despite obvious limitations, and strong hints for a publication bias which limits the evidence found in this meta-analysis, one can not ignore the fact that studies with positive effects of VA-E on survival of cancer patients are accumulating. Future studies evaluating the effects of *Iscador *should focus on a transparent design and description of endpoints in order to provide greater insight into a treatment often being depreciated as ineffective, but highly valued by cancer patients.

## Background

Complementary and alternative medicine (CAM) has become increasingly popular over the last decades. According to Bausell et al. [1], especially patients with chronic diseases increasingly seek for CAM-therapies. With a growing amount of health information in the internet, physicians and therapists and patients are often not prepared to judge provided information of CAM-health care approaches properly. Information dissemination of published evidence about the effectiveness of remedies and therapies therefore forms a necessary basis for shared-decision making for patients and practitioners.

In Europe, extracts from *Viscum album *(VA-E), the European white-berry mistletoe, are widely used to treat patients with cancer, but also with arthrosis, hypertension, arteriosclerosis, diabetes etc. [2]. Historically, the intentions of mistletoe uses were manifold and conflicting in several cases (i.e., swellings or tumours, epilepsy, diseases of spleen and liver, labour-pains, 'weakness of the heart' and oedema, eczema, ulcers of the feet, burns, and granulating wounds) [3]. In 1920, mistletoe extracts were introduced for the first time as a cancer treatment by Rudolf Steiner (1861-1925) [4], founder of anthroposophy. He recommended a drug extract produced in a complicated manufacturing process combining sap from mistletoe harvested in the winter and summer [5]. Based on his recommendations, several Anthroposophic doctors have treated their cancer patients with these extracts within the last century, but published - if at all - just some field reports. Moreover, pharmacological studies on the suggested anti-tumour effects of were completely lacking.

Meanwhile, clinical evaluations of mistletoe as an adjuvant cancer treatment have expanded. During the 1960s, Vester and Nienhaus isolated carcinostatic protein fractions which were recognized later as the cytotoxic viscotoxins and mistletoe lectins [6]. Recent scientific research has confirmed the folklore with evidence that mistletoe extracts (1) induce apoptosis, (2) stimulate immunocompetent cells, and (3) protect the DNA of mononuclear cells (for review see [7,8]). Several experiments using tumour-bearing animals showed impressive reduction of tumour growth and/or increased survival with the application of mistletoe therapy (for review see [7-9]). The cytotoxic effects were clearly related to the viscotoxins and cytotoxic mistletoe lectins, while the immuno-modulating effects were ascribed to the mistletoe lectins, poly-/oligosaccharides, viscotoxins and several other components (reviewed in [7-10]).

Results from *in vitro *studies and animal models indicate that the direct application of VA-E and their specific components (i.e., the cytotoxic mistletoe lectins) results in a destruction of tumours and metastases, and in an increased survival of the animals. Thus one may conclude that the intratumoural injection might be an effective route of application. Nevertheless, mistletoe extracts are recommended (and authorized) to be applied subcutaneously and not intratumourally.

Moreover, there are several whole plant extracts from *Viscum album *on the market which differ with respect to the extraction process and thus relative proportions of their constituents (i.e., the Anthroposophic manufacturers mix the mistletoe saps of the summer and winter harvest by complicated procedures: *Abnoba *extracts are produced by aqueous maceration of fresh plant material; *Helixor *and *Isorel *extracts are produced by cold water extraction; *Iscador *extracts are produced by fermentation of the plant material; *Iscucin *is produced in accordance with the German Homeopathic Pharmacopoeia; the phytotherapeutic companies produce the *Eurixor *and *Lectinol *extracts by an aqueous extraction of the fresh plant material harvested from poplars during the winter season) [11].

Due to this diversity of mistletoe products and their proportions of pharmacologically relevant constituents, the interpretation of clinical studies is difficult. Consequently it is not too surprising that ante ceded reviews on the clinical effects of mistletoe extracts in cancer patients, which summarizes a mixture of studies with different designs and plant extracts used, are conflicting in their results [12-17].

For this review we decided to focus on the survival associated with the most commonly used mistletoe extracts which is covered by a large spectrum of published studies, the fermented plant extract *Iscador *(WELEDA AG, Switzerland). This whole plant extract is produced from fresh leafy shoots and fruits of the summer and winter harvest, is rich on mistletoe lectins and viscotoxins [11,18], and is recommended to be applied 2-3 times per week subcutaneously. We intended to determine the effectiveness of the VA-E *Iscador *in the treatment of patients with cancer with respect to survival.

## Methods

### Search strategy

We searched several databases such as PubMed/Medline, the Excerpta Medica Database (EMBASE), the Cochrane Library, database of DIMDI (Deutsches Institut für Medizinische Dokumentation und Information) and CAMbase for clinical studies focusing on survival of cancer patients using *Iscador *extracts. Separate search terms were "Iscador" and "study", "mistletoe" and "study", and "Viscum" and "study". Finally we asked several experts for gray literature not listed in the above mentioned databases, checked the reference lists of relevant articles and authors, and contacted the manufacturer of mistletoe preparation. The complete search was performed between February and April 2008.

### Selection criteria

Inclusion criteria were controlled clinical studies (at least historic or literature) on parameters associated with survival in cancer patients treated with the VA-E *Iscador*, published in English or German language journals. We excluded field reports, case series, case reports, studies without any control group, abstracts which proceeded a full length publication, translations of already published manuscripts, double publication of similar data (exception is the presentation of further data), internal reports and unpublished manuscripts. In a few cases we had to exclude studies because of the simultaneous usage of the fermented extract *Iscador *and the aqueous extract *Helixor*.

### Analysis of data

Two review authors independently assessed trials for inclusion in the review. They took part in the extraction of data and assessment of study quality and clinical relevance. Disagreements were resolved by consensus. We graded the methodological quality of the studies by the following checklist (rater assessment): Adequate description of the study design (retrospective, prospective, retrolective, multicenter study etc.), subject assembly process (randomization, matched pairs, etc.), comparability of groups, description of drop outs, allocation concealment (analysis of concealed treatment allocation was difficult because most studies did not provide sufficient data to judge - either there were no statements or information are at least unclear), description of the intervention (dosage and duration of VA-E application), description of statistical analysis, external validity (representative patients, relevant therapeutic concepts, generalization of results). However, we did not explicitly refer to rating scores such as the JADAD, because blinding of VA-E application is difficult and, due to ethical reasons, rejected by several medical doctors. Thus, 2 out of 5 criteria of the JADAD score were not applicable for these studies; nevertheless, randomization as a criterion was assessed, also dropouts.

The reporting of the results adhered, if possible and appropriate, to the MOOSE guidelines [19], which involve recommendation for the description of study selection, presentation of results (including a table with descriptive information for each included study), and discussion of biases, consideration of alternative explanations for observed results etc.

### Data extraction

If a trial was found to be eligible, assessments of its methodological quality were done independently by two reviewers (AB, TO) and recorded on a pre-especially designed data form together with the basic trial data and the extracted results. Allocation concealment was assessed in accordance with the Cochrane guidelines:

A = adequate (telephone randomization or using consecutively numbered, sealed, opaque envelopes)

B = uncertainty about the concealment (method of concealment is not known).

C = inadequate (e.g. alternate days, odd/even date of birth, hospital number)

Disagreements on methodological quality ratings were discussed by both assessors until they reached a consensus.

Data were independently extracted by two persons (AB, TO) and independently entered into a data form which was especially designed for trials on VA-E by a third person (CR). If the data entries differed, both reviewers were contacted to recheck the publications and were forced to come to a consensus, which could be reached in all cases.

Data on the following topics were:

* Details of the publication (first author, country, year, journal)

* Details on the dosage and application of *Iscador*

* Type, name, dosage and application of the control therapy/alternative therapies

* Grading and location of cancer

* Age and gender distribution of patients

* Methodological quality of the study (see above)

* Outcome(s): Survival (median survival, overall survival, 3-, 5- or 10-year survival etc.)

### Statistical analysis

All data were separately analyzed for (a) placebo controlled trials, (b) actively controlled trials, and (c) trials where patients of the control group received only standard care but no extra treatment. Control groups where patients were "insufficiently treated" with the VA-E (i.e., < 3 packages within several months/years) were counted as having received no extra treatment. In one study, the patients received a glycopeptide preparation from sheep spleens (Polyerga Neu) versus *Iscador *versus a complex of B-vitamins which was regarded as a placebo, but was regarded as an "alternative therapy" in our analysis. Active controls used in the studies were interferon alpha2b or interferon gamma, Bacillus Calmette-Guérin (BCG), Vitamin B complex, or radiation.

Outcome data were extracted as hazard ratios (HR), their logarithm, and the respective standard errors. If HRs were not given, we assumed that the overall survival was exponentially distributed. This allowed us to estimate hazard ratios, even if only median survival times or survival rates were given in the publication. HR < 1 indicate superiority of *Iscador*, HR > 1 indicate superiority of the control condition.

The association between study size and trial results was graphically displayed in funnel plots, by plotting HRs on the horizontal axis (in a logarithmic scale) against their standard errors - or against the total patient numbers - on the vertical axis [20]. Funnel plots are adequate instruments to detect small study size effects, including publication bias. In the absence of bias, results from small studies should scatter widely at the bottom of the graph, with the spread narrowing among larger studies. Publication bias may lead to asymmetrical funnel plots. Moreover, the asymmetry of the funnel plot was further explored by a weighted linear regression analysis (meta-regression) which modelled the log HR as a function of its standard error [21]. Weights were chosen inversely to the squared standard error. From this model, the asymmetry coefficient (AC) was estimated as the slope of the regression line.

Heterogeneity between trials was assessed by standard χ^2^-tests and the I^2 ^coefficient [22] which measures the percentage of total variation across studies due to true heterogeneity rather than chance.

Overall estimates of the treatment effect were obtained from random effects meta-analysis [23]. Additionally, from meta-regression a predicted HR was obtained for trials with a standard error as small as the smallest observed standard error of all included trials.

The extent to which study-level variables were associated with log HRs was investigated by fitting multivariable meta-regression models. The following variables were considered: standard error of log HR, tumour localization (breast, stomach, lung, colon, ovary, corpus, skin yes/no), randomization (yes/no), matched-pair comparison (yes/no) - due to the fact that all matched-pair studies were from the same source.

## Results

We found 52 publications on the clinical effects of *Iscador *usage on survival of cancer patients (descriptive details and references in the additional file 1). Some reports describe data on different sets of patients and/or tumour stages or localization (strata), or different study designs within the same report.

**Figure 1:**
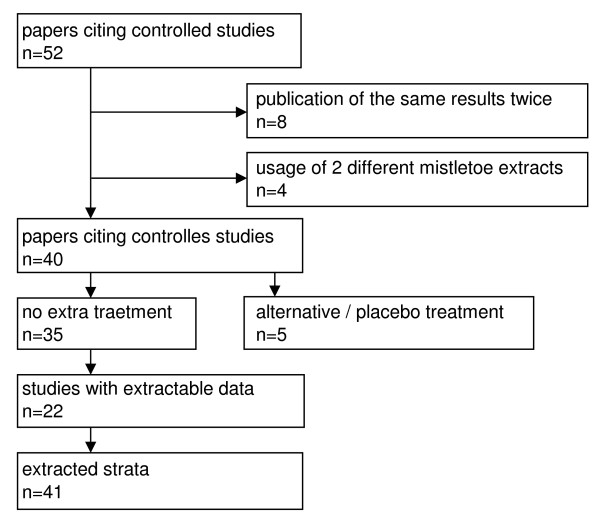
**Study selection process**.

As depicted in Figure 1, eight studies citing the same results twice in different papers and thus were excluded, four studies were excluded because of the usage of two different mistletoe extracts (*Helixor *and/or *Iscador*) which were not shown separately. Five studies used alternative or placebo controls (these data were presented independently), and 35 studies investigated effects of *Iscador *versus no extra treatment.

### Iscador *versus *no extra treatment

Forty one strata (i.e., localization, stages, lymph nodes etc.) found in 22 studies (Figure 1, additional file 1) provided enough data to extract HRs and their standard errors. Twelve studies were prospective in design, five were randomized, and ten had a matched-pair design. According to the nature of the control group, no study was blind. The oldest study dated back to 1963, the most recent was published in 2008. The number of patients enrolled varied considerably from 17 to 1,719, overall 3,388 patients were treated with *Iscador *and 7,253 patients served as controls. The studies included in this meta-analysis were of moderate or even poor quality, as indicated by randomization, matched pair building, blinding, multicenter, description of dropouts etc. (additional file 1).

**Figure 2:**
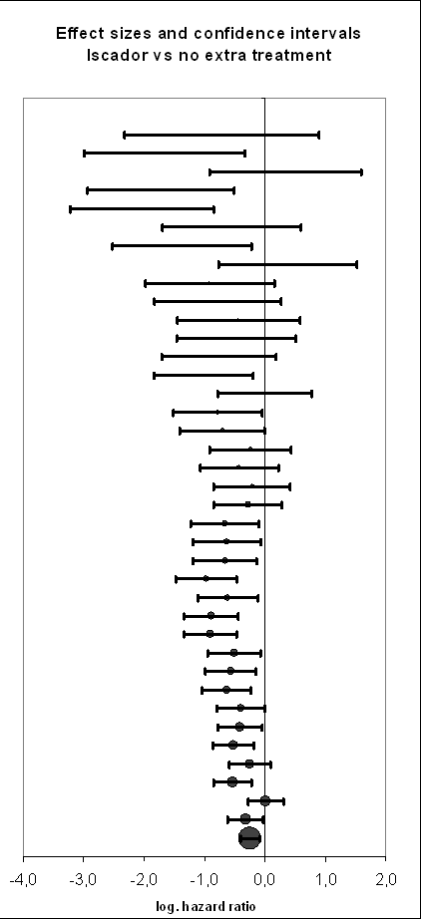
**Standardized treatment effects (HR and CI) of Iscador *versus *standard treatment (the size of circles represents the weight of the study/strata in meta-regression)**. Depicted are the results of 41 strata.

**Figure 3:**
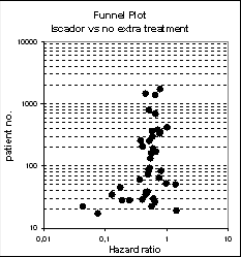
**Funnel plots with respect to standard errors (the line represents the regression line from meta-regression)**.

**Figure 4:**
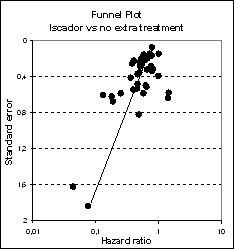
**Funnel plots with respect to total patient numbers**.

As shown in figure 2, the majority of studies reported positive effects in favour of the *Iscador *application. Heterogeneity of study results was moderate (I^2 ^= 38.3%, p < 0.0001). Two very small studies showed a very huge effect in favour of *Iscador*, but even when these two studies were discarded, the funnel plots were considerably skewed (figures 3 and 4), a notion which is corroborated by statistical means (AC = -1.3, CI: -1.9 to -0.6, p < 0.0001).

A random effect meta-analysis estimated the overall hazard ratio at HR = 0.59 (CI: 0.53 to 0.66, p < 0.0001). Simple meta-regression yielded a predicted HR = 0.74 (CI: 0.66 to 0.82, p < 0.0001). In multivariable meta-regression tumour localization generally was not significantly associated with better or worse study outcome (table 1), but lung cancer studies showed slightly better outcome than others (ratio of HRs: 0.56, CI: 0.00 to 1.10, p = 0.095). However, randomized studies showed less effects than non-randomized studies (ratio of HRs: 1.24, CI: 0.79 to 1.92, p = 0.35), and matched-pair studies gave significantly better results than others (ratio of HRs: 0.33; CI: 0.17 to 0.65, p = 0.0012).

**Table 1:** Multivariable meta-regression of tumour localization

Tumour localization	Hazard ratio	Confidence interval	P-value
Breast	1.24	0.84 to 1.85	0.277

Stomach	1.26	0.80 to 1.99	0.325

Lung	0.58	0.00 to 1.10	0.095

Colon	1.24	0.84 to 1.85	0.277

Ovary	1.27	0.81 to 1.98	0.298

Corpus	1.03	0.70 to 1.51	0.879

Skin	1.35	0.88 to 2.05	0.165

### Iscador *versus *placebo

A randomized placebo-controlled clinical trial was undertaken in 224 patients with advanced lung cancer [24]. Here, the patients received a glycol-peptide preparation from sheep spleens (Polyerga Neu) versus *Iscador*; however, there were no differences between median survival times (9.1 and 9.0 months) could be found.

### Iscador *versus *alternative therapies

Some trials (respectively strata) were identified which employed an active treatment as a control arm [24-28], three of them were randomized controlled trials [24-26,26].

Study sizes ranged from 46 to 227 patients, overall 462 patients were treated with *Iscador*, 450 patients with the alternative therapy.

Heterogeneity of study results was moderate and not statistically significant beyond chance (I^2 ^= 36.6%, p = 0.15). The funnel plots were not skewed (AC = 0.2, CI: -1.7 to 2.1, p = 0.86).

No treatment effect could be shown in a random effects meta-analysis (HR = 0.95, CI: 0.81 to 1.12, p = 0.56). Similar results were obtained from simple meta-regression (predicted HR = 0.94, CI: 0.71 to 1.24, p = 0.66). Multivariable meta-regression showed that randomized studies, however, gave significantly worse results than non-randomized studies (ratio of HRs: 3.20; CI: 1.16 to 8.85, p = 0.0247).

### Iscador *versus *no treatment

One non-randomized study compared 81 Iscador treated breast cancer patients with 30 patients who received an insufficient treatment (< 4 *Iscador*) packages within 5 years) which was assigned as "no treatment" [29]. Response rates were 74% and 46% respectively, which can be translated into a HR = 0.39 (CI: 0.20 to 0.77; p = 0.0068).

## Discussion

In this meta-analysis, we investigated the effect of *Iscador *on the survival rates of cancer patients. We were able to demonstrate that adjuvant treatment with *Iscador *was associated with a significant overall enhancement of survival rates. In particular, the benefit of *Iscador *with respect to survival was clearly seen in matched pair studies, but notably less in randomized trials. We also identified the design of the study as a source of variation among the studies on the survival of cancer patients using a meta-regression approach.

With the rapid evolution of both surgical and chemotherapeutical therapies, the question of survival of cancer patients is extensively debated. While prognosis of some cancer entities, i.e. recurrent breast cancer, has improved within the last decades [30], other cancers only tend to be moderately impressive, i.e. gall bladder cancer [31]. As a consequence different oncological treatment regimes were test - and not all of them were convincing. Other attempts were the application of low-dose chemotherapeutic regimen in distinct patients, i.e. advanced unresectable hepatocellular carcinoma, which were quite effective without worsening the quality of life of the patients [32]. Thus, more tolerable treatment regimes for cancer or the combination of conventional chemotherapy with adjuvant therapies gain more and more attention in the light of the primary aim to improve the situation of cancer patients. From that point of view it is worth to mention that a similar meta-analysis investigated the effects of VA-E used as an adjuvant in the treatment of cancer patients showed moderate improvements on quality of life (Büssing et al., in preparation); however, the methodological quality of these studies was quite heterogeneous.

Quite similar, the studies included in this meta-analysis were of moderate or even poor quality (additional file 1). Although studies in this area face a number of unique challenges and therefore might be difficult to conduct, most of the studies were on a low level of documentation quality. In particular in older studies, traceability and transparency was missing and thus has to be considered when looking at our results.

The prospect of randomizing patients to an adjuvant mistletoe treatment often raises ethical debates and negatively impacts the feasibility of studies [33]. Not surprisingly, the published randomized trials (not only in this area of research) are often small and consist of a selected patient populations compared to observational studies [34]. Furthermore, due to limited resources, a properly conducted long-term follow-up in survival is often omitted.

Although the benefit of adjuvant mistletoe treatment has been demonstrated in some randomized and observational studies, a comprehensive meta-analytical approach like the present one has not been previously conducted. In 2003, Ernst et al. [12]published a systematic review on randomized clinical trials (RCTs) and stated that "statistical pooling was not possible because of the heterogeneity of the primary studies. Therefore a narrative systematic review was conducted." We can confirm the heterogeneity of studies on the clinical effects of VA-E, but nevertheless were able to extract data from 41 studies which provided enough data to calculate HRs and their standard errors. Ernst et al. [12] stated that the weaker studies implied benefits of VA-E, particularly in terms of quality of life, while none of the methodologically stronger studies were able to verify a benefit with respect to survival or quality of life. A Cochrane Review of Horneber et al. [13] published in 2008 analyzed RCTs on various mistletoe extract preparations. The authors found weak evidence from RCTs that VA-E application impacts the survival of cancer patients, but that it could be effective with respective to quality of life during chemotherapy for breast cancer. In contrast, Kienle and Kiene provided a different point of view [15,16]. In their systematic review of RCTs from 2003 they identified 23 studies which met their inclusion/exclusion criteria [15]. Most of these studies reported statistically significant positive outcomes (or at least positive trends) for survival or tumour remission and quality of life, while several studies reported no effect on survival, recurrence, remission and QOL; just one study showed a negative trend for disease-free-survival [15]. Also Kienle et al. agreed that the methodological quality of several studies was "far below the standard that is today regarded as optimal or necessary" [15]. In 2007, Kienle and Kiene [16] published a systematic review of prospective clinical trials on Anthroposophic mistletoe extracts and identified 16 randomized and 9 non-randomized trials. Among them, 8 of 17 trials stated a significant benefit in favour of the VA-E with respect to survival; remission of tumour and malignant effusion in 2 of 4 controlled trials; for quality of life in 3 of 5 studies, and for quality of life and reduction of side effects of cytoreductive therapies in 5 of 7 trials [16]. They concluded that the best evidence for efficacy of VA-E exists for improvement of quality of life and reduction of side effects of cytotoxic therapies, while the survival benefit was a matter of critique [16].

Methodological quality of studies on the clinical effects of VA-E has improved over the last years; however, it is not surprising that particularly the older studies did not meet the current methodological standards. Indeed, most of the identified studies did not report data on compliance and completeness of follow up, intention to treat analysis was rarely mentioned, clear description of *Iscador *usage (duration, dosage) was reported in just a few cases, etc. For this analysis we did not judge RCTs as methodological 'better' that non-randomized; each methodological design has its unique weakness. It can not be ignored that results from RCTs, despite of their higher internal validity, often have a lower external validity. Particularly the studies of Grossarth-Maticek [35-43] tried to address this problem and used a mixed design, i.e. they combined a randomized matched-pair study with a non-randomized matched pair study within the same trial. In most cases, both study designs exhibited similar results. Although we were aware that it is quite problematic to separate these nested RCT from the non-RCT, we nevertheless decided to do so. We noticed that the survival observed in randomized studies was lower than in the non-randomized studies, and that matched-pair studies gave significantly better results than others. However, several positive reports (and strata) were from the same origin [35-43] and thus had the same methodological problems. These studies had a matched pair design, either with or without randomization; the description of the methodology was good, the discussion of potential bias factors was profound. It is obvious that the strict matching process significantly affected the number of patients enrolled in the evaluation (all studies had sample sizes of <200 subjects). Potential bias factors which might contribute to the overall positive effects described in the studies of Grossarth-Maticek et al. [35-43] were discussed in detail by the authors themselves [35,39], i.e., selection bias and loose matching, etc. However, because these studies started in 1973, several relevant study objectives were not available, i.e., exact dates of first diagnosis, operation, initial and follow up-data assessments and matching, socio-economic status, social support, spirituality etc. In these studies, attrition bias was less important because with the drop out of any study patient, the matching partner was also excluded and thus the balance of the groups was not severely affected [35,39]. Altogether, the internal validity of these study results was limited by selection bias and confounding. Moreover, there was no written protocol and no statistical hypotheses, the sample sizes were in most cases very small, and no sample size calculation. Another intriguing fact could be that the non-randomized studies of Grossarth-Maticek's group nevertheless might have a lower external validity (generalisability), because the inclusion and exclusion criteria were not very precise and not all of them explicitly formulated in advance [35,39]. Moreover, apart from the matching criteria, there were no explicit procedures for building pairs. The most important fact was raised by the authors themselves [35,39,43], as they can not exclude the possibility that preferentially patients with a good prognosis were enrolled, since patients from both groups who died shortly after the diagnosis would not have entered the study.

## Limitations

A limitation of this meta-analysis is that the pooled estimates are driven by quite heterogeneous data, although our estimate of I^2 ^= 38.3% laid below the critical boundary of 0.5 recommended by Higgins & Thompson [44]). Nevertheless, as already mentioned, it has to be taken into account that our study pool is a composite of quite different studies. As long-term survival by definition can only be obtained from studies which were conducted a number of years ago, we also included older studies employing treatment techniques which are now considered to be outdated. Stratified analysis however suggests that start year of the study does not affect the pooled effect estimates.

This meta-analysis was restricted to published studies. Although we tried to be as comprehensive as possible in our search for studies, the funnel plot is quite skewy and indicates a significant proportion of publication bias, which limits the evidence found in this meta-analysis. In particular the pooled effect estimates are mainly driven by the study type (i.e., matched-pair design). This phenomenon has already been recognized in other areas of research and thus is not due to the specific type of intervention presented here [45]. Moreover, this does not argue against the possibility that the patients treated with *Iscador *had better survival rates, but should be an indicator to ask for possible confounder or moderator variables which remain to be identified.

## Conclusions

In conclusion, pooled analysis of clinical studies suggests that adjuvant treatment of cancer patients with *Iscador *is associated with a reduction in mortality rates. Having in mind the limitations found here, future studies evaluating the effects of *Iscador *should continue to address this question, with a particular focus on a transparent design and description of endpoints in order to provide greater insight into a treatment often being depreciated as ineffective. The information and considerations from this analysis should be taken seriously not only for a better study quality but also to provide the best possible care for cancer patients.

## Abbreviations

CAM: complementary or alternative medicine; HR: hazard ratio; RCT: randomized clinical trials; SMD: standardized mean differences; VA-E: *Viscum album *extracts.

## Competing interests

AB and TO received financial support with a grant of Hiscia - Verein für Krebsforschung, Arlesheim (Schweiz). The authors have no competing interests, and were free to interpret the data according to a strict scientific rationale.

## Authors' contributions

AB and TO initiated and oversaw the project, wrote the paper, contributed to the project design, data analysis, interpretation, and the writing of the paper. CR contributed to documentation and data extraction.

## Pre-publication history

The pre-publication history for this paper can be accessed here:

http://www.biomedcentral.com/1471-2407/9/451/prepub

## Supplementary Material

Additional file 1 **Overview on identified clinical studies and strata**. All references details were given in this additional file too. Click here for file
