# Supplementary material for: Survival of cancer patients treated with mistletoe extract (Iscador): a systematic literature review
Source: BMC Cancer. 2009 Dec 18;9:451. doi: 10.1186/1471-2407-9-451 (PMC2804713; doi:10.1186/1471-2407-9-451)
Supplement: Additional file 1 — Overview on identified clinical studies and strata. All references details were given in this additional file too. [file 1471-2407-9-451-S1.DOC]

## Additional file 1 - Overview on identified clinical studies and strata

| **Author** | **Year** | **Tumor**  **localization** | **Study design**  **of all studies** | | | | **design of studies enrolled for statistical analysis** | | |
| --- | --- | --- | --- | --- | --- | --- | --- | --- | --- |
|  |  |  | **control** | **design** | **random.** | **matched pairs** | **Blinding** | **Multi-center** | **Description Drop-Outs** |
| Augustin [1] * | 2005 | Skin | PGD | retrolec. | NR | N-MP | no | **yes** | **yes** |
| Bock [2] * | 2004 | Breast | PGD | retrolect. | NR | N-MP | no | **yes** | unclear |
| Dold [3] | 1991 | Lung | PGD | prosp. | **rand** | N-MP | - | - | - |
| Eggermont [4] | 2001 | Skin | PGD | prosp. | **rand** | N-MP | - | - | - |
| Fellner [5-7] * | 1966 | Cervix | PGD | prosp. | NR | N-MP | no | no | no |
| Feuchtinger[8,9] | 1977 | Skin | Lit | retro. | - | - | - | - | - |
| Grossart-Maticek [10] * | 2004 | Various | PGD | prosp. | NR | **MP** | no | **yes** | unclear |
| Grossart-Maticek [11-13] * | 2006 | Breast | PGD | prosp. | **rand** | **MP** | no | **yes** | yes |
| Grossart-Maticek [12,14,15] | 2006 | Breast | PGD | prosp. | NR | **MP** | - | - | - |
| Grossart-Maticek [16] | 2006 | Breast | PGD | prosp. | **rand** | **MP** | - | - | - |
| Grossart-Maticek [17] | 2006 | Breast | PGD | prosp. | NR | **MP** | - | - | - |
| Grossart-Maticek [18] | 2007 | Ovary | PGD | prosp. | **rand** | **MP** | - | - | - |
| Grossart-Maticek [18] | 2007 | Ovary | PGD | prosp. | NR | **MP** | - | - | - |
| Grossart-Maticek [19] */* | 2007 | Cervix | PGD | prosp. | NR | **MP** | no | **yes** | yes |
| Grossart-Maticek [20] * | 2007 | Cervix | PGD | prosp. | **rand** | **MP** | no | **yes** | yes |
| Grossart-Maticek [21] | 2007 | Skin | PGD | prosp. | **rand** | **MP** | - | - | - |
| Grossart-Maticek [21] * | 2007 | Skin | PGD | prosp. | NR | **MP** | no | **yes** | yes |
| Grossart-Maticek [22] */* | 2008 | Corpus uteri | PGD | prosp. | NR | **MP** | no | **yes** | no |
| Grossart-Maticek [23] */* | 2008 | Corpus uteri | PGD | prosp. | **rand** | **MP** | no | **yes** | **yes** |
| Günczler [24] | 1962 | Breast | Lit | retro. | - | - | - | - | - |
| Günczler [25,26] */*/* | 1968 | Stomach | PGD | retro. | NR | N-MP | no | no | yes |
| Günczler [27] */* | 1969 | Breast | historic | retro. | - | - | no | no | unclear |
| Hassauer [28] * | 1979 | Ovary | Lit | retro. | - | - | no | no | unclear |
| Hellan [29] | 1995 | Colorectal | PGD | retro. | NR | N-MP | - | - | - |
| Hoffmann [30] | 1979 | Colorectal | PGD | retro. | NR | N-MP | - | - | - |
| Hoffmann [31] | 1980 | bladder | PGD | retro. | NR | N-MP | - | - | - |
| Hoffmann [32] */*/* | 1982 | Breast | Lit | retro. | - | - | no | no | no |
| Kjaer [33,34] | 1984 | Kidney | historic | prosp. | - | - | - | - | - |
| Kleeberg [35] | 2004 | Skin | PGD | prosp. | **rand** | N-MP | - | - | - |
| Krause [36] | 1983 | Lung | historic | retro. | - | - | - | - | - |
| Koch [37] | 1980 | Breast | Lit | retro. | - | - | - | - | - |
| Leroi [38] */* | 1977 | Breast | PGD | retro. | NR | N-MP | no | no | no |
| Leroi [39] | 1978 | Colorectal | PGD | retro. | NR | N-MP | - | - | - |
| Leroi [40] | 1979 | Colorectal | PGD | retro. | NR | N-MP | - | - | - |
| Leroi [41] | 1975 | Breast | PGD | retro. | NR | N-MP | - | - | - |
| Leroi [42] */* | 1982 | Ovary | Lit | retro. | - | - | no | no | no |
| Majewski [43] */*/*/* | 1963 | Ovary | PGD | retro. | NR | N-MP | no | no | yes |
| Salzer [44,45] */*/*/*/* | 1975 | Lung | PGD | retro. | NR | N-MP | no | no | yes |
| Salzer [46] */*/* | 1978 | Lung | PGD | retro. | NR | N-MP | no | no | no |
| Salzer [47,48] * | 1983 | Stomach | PGD | prosp. | **rand** | N-MP | no | no | unclear |
| Salzer [49] | 1991 | Lung | PGD | prosp. | **rand** | N-MP | - | - | - |
| Schaefermeyer [50] | 1998 | Pancreas | Lit | retro. | - | - | - | - | - |
| Schreiber [51] * | 1984 | Ovary | Lit | retro. | - | - | no | no | unclear |
| Schreiber [51] * | 1984 | Ovary | PGD | retro. | NR | N-MP | no | no | unclear |
| Schuppli [52] | 1990 | Skin | PGD | retro. | NR | N-MP | - | - | - |
| Wagner [53] | 1996 | Ovary | Lit | retro. | - | - | - | - | - |

PLG – parallel group; Lit – literature control; NR – no randomization; rand – randomization; MP – matched pairs; N-MP – no matched pairs; prosp. – prospective; retro. – retrospective; retrolec. – retrolective

* study/strata suited for data extraction

**Table references**

1. Augustin M, Bock PR, Hanisch J, Karasmann M, Schneider B: **Safety and efficacy of the long-term adjuvant treatment of primary intermediate- to high-risk malignant melanoma (UICC/AJCC stage II and III) with a standardized fermented European mistletoe (Viscum album L.) extract. Results from a multicenter, comparative, epidemiological cohort study in Germany and Switzerland.** *Arzneimittelforschung* 2005, **55:** 38-49.

2. Bock PR, Friedel WE, Hanisch J, Karasmann M, Schneider B: **Wirksamkeit und Sicherheit der komplementären Langzeitbehandlung mit einem standardisierten Extrakt aus Europäischer Mistel (Viscum album L.) zusätzlich zur konventionellen adjuvanten onkologischen Therapie bei primärem, nicht metastasiertem Mammakarzinom.** *Arzneimittelforschung / Drug Research* 2004, **54:** 456-466.

3. Dold U, Edler L, Mäurer HCh, Sakellariou B, Trendelenburg F, Wagner G: *Krebszusatztherapie beim fortgeschrittenen nicht-kleinzelligen Bronchialkarzinom*. Stuttgart: Georg Thieme Verlag; 1991.

4. Eggermont AM, Kleeberg UR, Ruiter D, Suciu S: **European Organization for Research and Treatment of Cancer Melanoma Group. Trial experience with more than 2000 Patients, evaluating adjuvant treatment with low or intermediate doses of interferon Alpha- 2b.** In *American Society of Clinical Oncology. Educational Book*. Edited by Perry MC. Alexandria, USA: 2001:88-93.

5. Fellmer KE, Fellmr Ch: **Nachbehandlung bestrahlter Genitalkarzinome mit dem Viscum-album-Präparat Iscador®.** *Krebsarzt* 1966, **21:** 174-185.

6. Fellmer KE: **Nachbehandlung bestrahlter Genitalkarzinome mit dem Viscum-album-Präparat Iscador® zur Rezidivprophylaxe.** *Med Klin* 1967, **62:** 305-307.

7. Fellmer KE: **A clinical trial of Iscador.** *The British Homoeopathic Journal* 1968, **57:** 43-47.

8. Feuchtinger T: **Internistische Therapie ders malignen Melanoms.** *Mitteilungen der Behandlung maligner Tumoren* 1978, **10:** 12-18.

9. Feuchtinger T: **Ergebnisse der Internistischen Therapie des malignen Melanoms (Stadium II und III) mit Iscador®.** *Krebsgeschehen* 1982, **14:** 51-58.

10. Grossarth-Maticek R, Kiene H, Baumgartner SM, Ziegler R: **Synergieeffekte von Selbstregulation und Misteltherapie (Iscadr) auf die Überlebenszeit von Krebspatienten.** *Schweizer Zeitschrift für Ganzheitsmedizin* 2004, **16:** 81-89.

11. Grossarth-Maticek R, Kiene H, Baumgartner SM, Ziegler R: **Use of Iscador, an extract of European mistletoe (Viscum album), in cancer treatment: prospective nonrandomized and randomized matched-pair studies nested within a cohort study.** *Altern Ther Health Med* 2001, **7:** 57-72, 74.

12. Grossarth-Maticek R, Kiene H, Baumgartner SM, Ziegler R: **Verlängerung der Überlebenszeit von Krebspatienten unter Misteltherapie (Iscador).** *Schweizer Zeitschrift für Ganzheitsmedizin* 2001, **13:** 217-225.

13. Grossarth-Maticek R, Ziegler R: **Randomised and non-randomised prospective controlled cohort studies in matched-pair design for the long-term therapy of breast cancer patients with a mistletoe preparation (Iscador): a re-analysis.** *Eur J Med Res* 2006, **11:** 485-495.

14. Grossarth-Maticek R, Kiene H, Baumgartner SM, Ziegler R: **Use of Iscador, an extract of European mistletoe (Viscum album), in cancer treatment: prospective nonrandomized and randomized matched-pair studies nested within a cohort study.** *Altern Ther Health Med* 2001, **7:** 57-72, 74.

15. Grossarth-Maticek R, Ziegler R: **Randomised and non-randomised prospective controlled cohort studies in matched-pair design for the long-term therapy of breast cancer patients with a mistletoe preparation (Iscador): a re-analysis.** *Eur J Med Res* 2006, **11:** 485-495.

16. Grossarth-Maticek R, Ziegler R: **Prospective controlled cohort studies on long-term therapy of breast cancer patients with a mistletoe preparation (Iscador).** *Forsch Komplement Med (2006 )* 2006, **13:** 285-292.

17. Grossarth-Maticek R, Ziegler R: **Prospective controlled cohort studies on long-term therapy of breast cancer patients with a mistletoe preparation (Iscador).** *Forsch Komplement Med (2006 )* 2006, **13:** 285-292.

18. Grossarth-Maticek R, Ziegler R: **Prospective controlled cohort studies on long-term therapy of ovarian cancer patients with mistletoe (Viscum album L.) extracts iscador.** *Arzneimittelforschung* 2007, **57:** 665-678.

19. Grossarth-Maticek R, Ziegler R: **Prospective controlled cohort studies on long-term therapy of cervical cancer patients with a mistletoe preparation (Iscador).** *Forsch Komplement Med (2006 )* 2007, **14:** 140-147.

20. Grossarth-Maticek R, Ziegler R: **Prospective controlled cohort studies on long-term therapy of cervical cancer patients with a mistletoe preparation (Iscador).** *Forsch Komplement Med (2006 )* 2007, **14:** 140-147.

21. Grossarth-Maticek R, Ziegler R: **Wirksamkeit und Unbedenklichkeit einer Langzeitbehandlung von Melanompatienten mit einem Mistelpräparat (Iscador).** *Schweizer Zeitschrift für Ganzheitsmedizin* 2007, **19:** 325-332.

22. Grossarth-Maticek R, Ziegler R: **Randomized and non-randomized prospective controlled cohort studies in matched pair design for the long-term therapy of corpus uteri cancer patients with a mistletoe preparation (Iscador).** *Eur J Med Res* 2008, **13:** 107-120.

23. Grossarth-Maticek R, Ziegler R: **Randomized and non-randomized prospective controlled cohort studies in matched pair design for the long-term therapy of corpus uteri cancer patients with a mistletoe preparation (Iscador).** *Eur J Med Res* 2008, **13:** 107-120.

24. Günczler M, Salzer G: **Erfahrungen mit der eingeschränkten Radikaloperation und Iscador-Nachbehandlung beim Brustdrüsenkarzinom.** *Krebsarzt* 1962, **17:** 198-207.

25. Günczler M, Osika Ch, Salzer G: **Ergebnisse von Resektion und Nachbehandlung beim Magenkarzinom.** *Wiener Klinische Wochenschrift* 1968, **80:** 105-106.

26. Günczler M: **Ergebnisse und Erfahrungen in der Krebstherapie mit Iscador® I. Das Magencarcinom.** *Beiträge zu einer Erweiterung der Heilkunst* 1968, **21:** 188-195.

27. Günczler M, Salzer G: **Iscadortherapie in der Nachbehandlung operierter Carcinome.** *Österreichische Ärztezeitung* 1969, **24:** 2290-2298.

28. Hassauer W, Gutsch J, Burkhardt R: **Welche Erfolgsaussichten bietet die Iscador®-Therapie beim fortgeschrittenen Ovarialkarzinom?** *Onkologie* 1979, **2:** 28-36.

29. Hellan J, Danmayr E, Hellan M: **Stellenwert der Komplementärmedizin in der Behandlung onkologischer Patienten - dargestellt anhand des kolo-rektalen Karzinoms.** *Deutsche Zeitschrift für Onkologie* 1995, **27:** 85-94.

30. Hoffmann J: **Die Iscador®-Behandlung bei Lebermetastasen.** *Krebsgeschehen* 1979, **11:** 172-175.

31. Hoffmann J: **Behandlungsergebnisse bei den Blasenkarzinomen der Lukas-Klinik.** In *Behandlungsergebnisse beim Blasenkarzinom*. Edited by Rilling ES. Heidelberg: Verlag für Medizin Dr. E. Fischer; 1980:57-61.

32. Hoffmann J, Hajto T: **Iscador®-Behandlung beim metastasierenden Mammakarzinom.** *Krebsgeschehen* 1982, **14:** 70-75.

33. Kjaer M: **Misteltenbehandling af metastaserende nyrecaner. En fase II undersogelse.** *Ugeskr Laeger* 1988, **150:** 2352-2354.

34. Kjaer M: **Misteltoe (Iscador) therapy in stage IV renal adenocarcinoma. A phase II study in patients with measurable lung metastases.** *Acta Oncol* 1989, **28:** 489-494.

35. Kleeberg UR, Suciu S, Brocker EB, Ruiter DJ, Chartier C, Lienard D *et al*.: **Final results of the EORTC 18871/DKG 80-1 randomised phase III trial. rIFN-alpha2b versus rIFN-gamma versus ISCADOR M versus observation after surgery in melanoma patients with either high-risk primary (thickness >3 mm) or regional lymph node metastasis.** *Eur J Cancer* 2004, **40:** 390-402.

36. Krause F, Erkan F: **Adjuvante Iscador- Behandlung resezierter Bronchuskarzinome.** *Krebsgeschehen* 1983, **15:** 158.

37. Koch HL, Voß AC: **Zur Behandlung des Mammakarzinoms. Ein Vergleich zwischen postoperativer Strahlentherapie und Iscador- Langzeitbehandlung nach eingeschränkter Radikaloperation.** *Medizinische Welt* 1980, **31:** 1773-1775.

38. Leroi R: **Nachbehandlung des operierten Mammakarzinoms mit Viscum album.** *Helvetica Chirurgica Acta* 1977, 403-414.

39. Leroi R. Übersicht über die Behandlungsresultate bei Patienten mit fortgeschrittenem Rektumkarzinom. Mitteilungen der Behandlung maligner Tumoren 10[2], 19-23. 1978. Arlesheim, Verein für Krebsforschung.
Ref Type: Magazine Article

40. Leroi R: **Die Iscadorbehandlung bei inoperablen kolo-rektalen Tumoren.** *Krebsgeschehen* 1979, **11:** 163-165.

41. Leroi R: **Malignomtherapie mit neuen Iscador- Präparaten.** *Krebsgeschehen* 1975, **7:** 136-238.

42. Leroi R, Hajto T: **Die Iscadortherapie beim Ovarialkarzinom.** *Krebsgeschehen* 1982, **14:** 38-44.

43. Majewski A, Bentele W: **Über Zusatzbehandlung beim weiblichen Genitalkarzinom.** *Zentralblatt für Gynäkologie* 1963, **85:** 696-700.

44. Salzer G: **Klinischer Versuch zur Verbesserung des Schicksals "Radikaloperierter" Bronchuskarzinom-Patienten.** *Zeitschrift für Erkrankungen der Atmungsorgane* 1975, **142:** 127-131.

45. Salzer G: **Die adjuvante Behandlung operierter Bronchuscarcinome mit dem Mistelpräparat Iscador®.** In *Die Mistel in der Krebsbehandlung*. Edited by Wolf O. Frankfurt am Main: Vittorio Klostermann; 1985:113-123.

46. Salzer G, Havelec L: **Rezidivprophylaxe bei operierten Bronchuskarzinompatienten mit dem Mistelpräparat Iscador. Ergebnisse eines klinischen Versuchs aus den jahren 1969-1971.** *Onkologie Zeitschrift für Krebsforschung und -behandlung* 1978, **1:** 1-5.

47. Salzer G, Havelec L: **Adjuvante Iscador-Behandlung nach operiertem Magenkarzinom. Ergebnisse einer randomisierten Studien.** *Krebsgeschehen* 1983, **15:** 106-110.

48. Salzer G: **Prospektiv randomisierter Studie: Operiertes Magenkarzinom Adjuvante Behandlung mit Iscador.** *Deutsche Zeitschrift für Onkologie* 1988, **20:** 90-93.

49. Salzer G, Danmayr E, Wutzlhofer F, Frey S: **Adjuvante Iscador-Behandlung operierter nicht kleinzelliger Bronchuskarzinome.** *Deutsche Zeitschrift für Onkologie* 1991, **23:** 93-98.

50. Schaefermeyer G, Schaefermeyer H: **Treatment of pancreatic cancer with Viscum album (Iscador): a retrospective study of 292 patients 1986-1996.** *Complementary Therapies in Medicine* 1998, **6:** 172-177.

51. Schreiber K, Stumpf C: **Iscador in der postoperativen Therapie des Ovarialkarzinoms. Ergebnisse 24jähriger Therapie.** *Erfahrungsheilkunde* 1984, **33:** 349-358.

52. Schuppli R: **Die adjuvante Behandlung des malignen Melanoms mit Iscador c. Hg.** In *Krebs und Alternativmedizin II*. Edited by Jungi EWF, Senn H-J. Berlin - Heidelberg: Springer Verlag; 1990:84-87.

53. Wagner R: **Ovarial-Ca. und Misteltherapie.** *Der Merkurstab* 1996, **49:** 152-153.
